# A Diet Pattern Characterized by Sugar-Sweetened Beverages Is Associated with Lower Decision-Making Performance in the Iowa Gambling Task, Elevated Stress Exposure, and Altered Autonomic Nervous System Reactivity in Men and Women

**DOI:** 10.3390/nu15183930

**Published:** 2023-09-11

**Authors:** Kevin D. Laugero, Nancy L. Keim

**Affiliations:** 1 Obesity and Metabolism Research Unit, USDA/ARS/Western Human Nutrition Research Center 2, Davis, CA 95616, USA; nancy.keim@usda.gov; 2 Department of Nutrition, University of California, Davis, CA 95616, USA

**Keywords:** diet patterns, affective decision making, chronic stress, autonomic nervous system

## Abstract

The executive brain mediates and facilitates a set of cognitive functions, such as decision making, planning, self-regulation, emotional regulation, and attention. Executive dysfunction and related diseases are a rising public health concern. Evidence supports a link between nutritional factors and executive function (EF), but relatively little information exists about the relationship between diet patterns and this higher order cognitive ability. We and others have reported on the relationships between body weight regulation and affective decision making, as measured by performance in the Iowa Gambling Task (IGT). However, little is known about the relationships between performance in this decision-making task and whole diet patterns. In this study, we tested whether data-derived diet patterns based on energy-adjusted food intake data from the Block Food Frequency Questionnaire were associated with decision-making performance in the IGT. Secondarily, we examined the influence of these diet patterns on self-reported chronic stress exposure and heart rate variability, which is a marker of autonomic nervous system (ANS) activity. In prior studies, stress and ANS activity were shown to influence decision-making performance in the IGT. In this study, five distinct diet patterns were identified by cluster and factor analyses. A diet pattern best characterized by elevated sugar-sweetened beverage and added sugar consumption was associated with the lowest decision-making performance (*p* = 0.0049) and higher stress exposure (*p* = 0.0097). This same diet pattern was associated (*p* = 0.0374) with an IGT-affiliated decline in high-frequency HRV and an increase in low-frequency HRV, suggesting diet-induced ANS regulatory shifts in response to performing the EF task. Compared to the sugar-sweetened beverage diet pattern, diet patterns defined by more fruits/vegetables and low red meat (*p* = 0.0048) or higher omega-3 fatty acids and seafood (*p* = 0.0029) consumption were associated with lower chronic stress exposure. All outcomes were statistically adjusted for differences in BMI, age, sex, education level, and sensorimotor ability. Our findings provide new information that further supports the potential importance of whole diet patterns on cognitive disease prevention.

## 1. Introduction

Both diet and psychological stress are linked to executive brain function or decision-making abilities. Moreover, stress and executive brain function interact to affect dietary choice [1], and diet has effects on perceived stress and stress reactivity [2]. Most published research focuses on the influence of foods or nutrients on cognitive function, but the cognitive impact of diet patterns as a whole has more recently received significant attention. Similarly, relatively fewer controlled intervention studies have been conducted to determine the influence of whole diet patterns on psychological stress [3] or vice versa.

Our lab and others previously showed the performance in the affective decision-making task known as the Iowa Gambling Task to be associated in some way with body weight regulation and nutrition intervention responsiveness. For example, we found that performance in the Iowa Gambling Task was related to the magnitude of weight loss and salivary cortisol in a diet-induced weight loss intervention in overweight women [4]. These results, along with others [5,6,7], suggest that body weight regulation may be tied to executive functions that involve decision making about events that have emotionally or socially salient ramifications. These findings underscore the need to further examine higher cognitive pathways that may influence or be altered by diet, stress, and their interaction.

The Iowa Gambling Task (IGT) is one of the most extensively used decision-making tasks and is a computer-based card game developed to characterize the neurological basis for deficits in decision making in patients with lesions to the ventromedial pre-frontal cortex (vmPFC) and are otherwise normal in terms of IQ, measures of impulsivity, working memory, and basic reasoning [8]. This cognitive task probes vmPFC function by assessing a person’s ability to inhibit short-term rewards (high immediate payoff) when the long-term consequences are known to be detrimental (net loss). It is important to note that damage to other brain regions (e.g., amygdala) that play a role in emotional processing and directly communicate with prefrontal regions [9,10] is associated with impaired IGT performance [11]. The IGT was shown to be sensitive to impaired decision making in persons with bulimia and anorexia nervosa [12,13] and in overweight or obese individuals [5,6,7]. Less is known about the dietary influence on IGT performance, particularly whether diet patterns affect or associate with performance on this executive function task.

In this study, we evaluated whether diet patterns influenced performance in the Iowa Gambling Task in a relatively large cohort of men and women. We secondarily assessed whether the observed influence of diet pattern on this executive function task extended to self-reported chronic stress exposure and a biomarker of autonomic nervous system activity. We hypothesized that less healthy diet patterns would be associated with lower decision-making performance on the Iowa Gambling Task, higher chronic stress exposure, and altered autonomic nervous system activity around the gambling task.

## 2. Materials and Methods

### 2.1. Study Design and Subjects

Research participants were from two independent clinical research studies conducted at the United States Department of Agriculture Western Human Nutrition Research Center (WHNRC) in Davis, CA. The individual Metabolism and Physiological Signatures Study (iMAPS; ClinicalTrials.gov: NCT02298725) recruited pre- and post-menopausal women with overweight to obese BMIs who had <150 min/wk of physical activity and ≥1 cardiometabolic risk factor (n = 44) in an 8 week feeding intervention to test the impact of diets meeting the Dietary Guidelines for Americans on cardiometabolic risk factors. Cardiometabolic results from this study were previously published [14]. For this paper, secondary analyses of Iowa Gambling Task (IGT) performance, self-reported chronic stress exposure (Wheaton Chronic Stress Index), habitual diet (Block Food Frequency Questionnaire), and other relevant data were derived from a pre-intervention (baseline) test visit of the iMAPS study and combined with the same data types from research volunteers participating in the Nutritional Phenotyping Study (Phenotyping Study; ClinicalTrials.gov: NCT02367287). The Phenotyping Study [15,16] is a cross-sectional study that recruited a cohort of 393 generally healthy men and women living near Davis, CA. For the current study, after accounting for missing data, 349 participants of the total Phenotyping Study participants and 42 participants of the total iMAPS study participants were examined for associations between diet pattern and Iowa Gambling Task performance. For the chronic stress examination, 349 Phenotyping and 43 iMAPS participants were analyzed and, for heart rate variability assessment, 323 Phenotyping and 39 iMAPS participants were analyzed. Test visits for both studies were conducted at the WHNRC, and both studies were approved by the University of California Davis Institutional Review Board. For the combined sample used in this secondary analysis, there were 58% women and 42% men. The average BMI (kg/m^2^) for the total sample was 27.9 ± 0.2 (S.E.), and the average age (years) was 41.2 ± 0.7 (S.E.).

### 2.2. Measurements

#### 2.2.1. Iowa Gambling Task

A computerized version of the Iowa Gambling Task [4] was conducted in the afternoon between 2 and 4 PM. While participants were seated in front of the computer, the test administrator read a series of instructions. They were told that they were being loaned USD 2000 in play money and would be asked to make multiple card selections from four different decks on the screen: A, B, C, and D. Some card selections would result in a net gain of money while others would result in a net loss. It was further revealed that some decks in the game were better than others and that if they stayed away from the “bad” decks, they could ultimately win money. Subjects were left alone in the room for the duration of the test and were told to inform the administrator upon completion. Total scores were calculated by subtracting the overall number of choices from the disadvantage decks (A and B) by those of the advantageous decks (C and D). To account for any differences in sensorimotor performance, a motor screening task (MOT; Cambridge Cognition Ltd., Cambridge, UK) was administered to assess psychomotor speed. This test measures the individual’s speed and accuracy of response to colored crosses presented on a tablet screen. All statistical models used the information gained from this task to account for between-participant variation in sensorimotor performance.

#### 2.2.2. Wheaton Chronic Stress Inventory

The 51-item Wheaton Chronic Stress Inventory was used to assess chronic stress exposure. This self-report instrument examines the presence of chronic stressors related to work, relationship, and financial difficulties and ratings of impact [17]. Scores for each of the statements on the questionnaire were rated using a 3-point scale (0 = not at all true to 2 = extremely true) and the items were summed to obtain a total chronic stress exposure score, with possible scores ranging from 0 to 102. Participants filled out the questionnaire at the Western Human Nutrition Research Center under the same conditions (e.g., room, time, instructions). Examples of the questions include “Someone in your family or a close friend has a long-term illness or handicap”, “You are alone too much”, “Your rent or mortgage is too high”, and “You are trying to take on too many things at once”. Reliability was α = 0.87 in the current study.

#### 2.2.3. Habitual Diet

Habitual dietary intake was assessed in the Nutrition Phenotyping Study using the Block 2014 Food Frequency Questionnaire (FFQ) by NutritionQuest (2014). The Block 2005 FFQ was used in the iMAPS study. Food and nutrient consumption frequency over the past 12 months was examined using an electronic version of the FFQ which was administered in an interview with trained study personnel. The FFQ is a frequently used instrument and has been validated (e.g., [18].)

### 2.3. Autonomic Nervous System Activity

Heart rate variability (HRV) was used as an index of autonomic nervous system activity. Heart rate variability is the variation in time between heartbeats (inter-beat interval) and reflects coordinated activity in the sympathetic and parasympathetic nervous systems. HRV has been used to indicate health and well-being [19]. Moreover, activity in the autonomic nervous system is thought to influence decision making and performance in the Iowa Gambling Task (e.g., [20,21,22,23]). A hardware and software (BioLab 3.4.1) system provided by MindWare Technologies LTD was used to collect electrocardiogram-based inter-beat interval data for estimating heart rate variability metrics. Real-time data were monitored by wireless data transmission or captured locally on the collection device worn by the seated participants. Electrodes were placed on the body as predetermined by the manufacturer. A positive electrode was placed at the bottom left rib near the side, a negative electrode was placed on the right collar bone (clavicle), and the ground electrode was placed at the bottom right rib on the subject’s side. Men were asked to shave the placement sites according to a provided diagram before the study visit. Trained study personnel cleaned the skin at the application site using sterile alcohol prep pads and wiped it dry using gauze before placing the electrodes. Data were acquired using the MindWare system immediately before and during the Iowa Gambling Task. For standardization, data used to estimate heart rate variability metrics were taken from a 5 minute collection period immediately before the task and again for the first 5 min of the task. For the pre-task data collection, participants were asked to sit quietly during that dedicated data collection/rest period without access to mobile devices or reading materials. Data were processed and cleaned for artifacts using HRV software (HRV 3.2.11) from MindWare Technologies. Low frequency (LF) HRV, high frequency (HF) HRV, respiratory sinus arrhythmia (RSA), and the LF:HF HRV ratio were estimated and used for analysis.

### 2.4. Statistical Analysis

**Figure 1:**
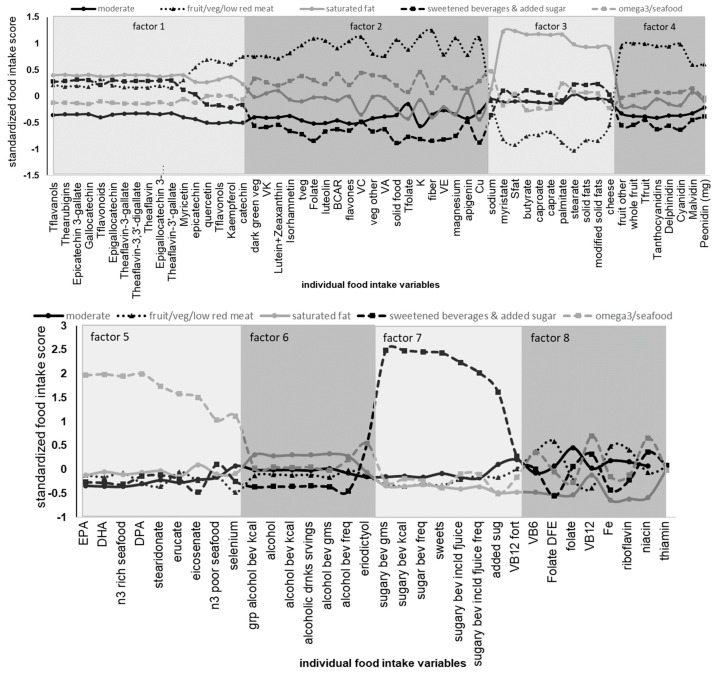

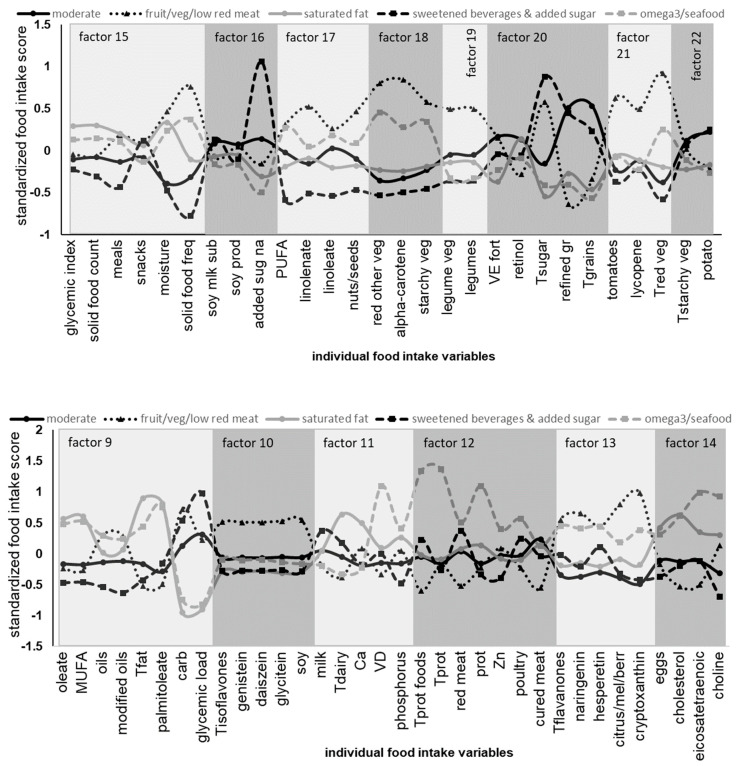

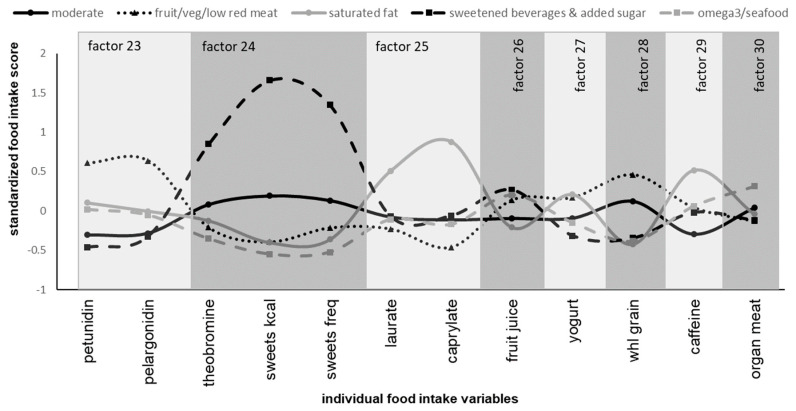
Diet patterns identified by cluster analysis can be described as generally moderate (solid black line, circles; N = 156), fruit/veg/low red meat (dotted black line, triangles; N = 91), saturated fat (solid gray line, circles; N = 78), sweetened beverages and added sugar (dashed black line, squares; N = 36), and omega 3 fatty acids and seafood (dashed gray line, squares; N = 42). To help quantifiably appreciate the food-related variables that characterized the cluster patterns, a factor analysis on all food-related variables used in the cluster analysis was conducted. Each factor is represented by those *x*-axis labels (individual food intake variables) and corresponding markers (squares/circles/triangles) within the shaded boxes.

All data were managed using the Research Electronic Data Capture (REDCap), which is a secure, web-based application for data collection for research studies hosted by the University California Davis Health System Clinical and Translational Science Center. SAS for Windows, version 9.4, was used for all statistical analyses. Tests for identifying clusters of diet quality patterns were conducted using a cluster analysis (Proc Fastclus) of FFQ food and nutrient variables. Five distinct diet pattern clusters were observed with the frequencies shown in Figure 1. Prior to cluster analysis, FFQ food and nutrient intake data were adjusted for total energy intake by dividing the FFQ intake data by total energy intake and multiplying by 1000. These energy-adjusted variables were then standardized using the (Proc Stdize) procedure before being subjected to the cluster analysis. To corroborate our findings based on the five distinct diet pattern clusters and to help quantifiably appreciate which food-related variables characterized the cluster patterns, we also used a factor analysis of the same standardized dietary data. This statistical method reduces the large number of dietary variables into fewer dietary variables (factors) that can help with interpretation of the diet patterns (clusters). Factors were determined using the PROC FACTOR procedure in SAS, wherein a principal components analysis and varimax rotation were used to identify noncorrelated factors. Thirty factors were retained based on a cutoff Eigenvalue of 1. A general linear model (SAS GLM procedure) was used to test the effects of diet pattern clusters on Iowa Gambling Task performance, log of Wheaton chronic stress score, and change in heart rate variability. Sex, age, BMI, education level, and sensorimotor score were considered as possible confounders, and were therefore included as independent variables in all statistical models. For testing the effects of diet patterns on IGT performance, the Wechsler Abbreviated Scale of Intelligence (WASI) test (Pearson Education, Inc., San Antonio, TX, USA) score was also included in the statistical model. We did not statistically adjust for multiple comparisons. We used the “effectsize” option to estimate affiliated effect sizes (eta-square) for the main effects of diet pattern on IGT, chronic stress, and HRV outcomes. Study type and study type by diet pattern cluster interaction terms were originally included in the statistical models to check for any influence of study type. The final models did not include these terms since they were not statistically significant and did not affect the association between diet pattern clusters and outcome variables. Adjusted mean (least square) differences between diet patterns were evaluated using the pdiff option. In all cases, *p* < 0.05 was considered to be statistically significant.

## 3. Results

**Figure 2:**
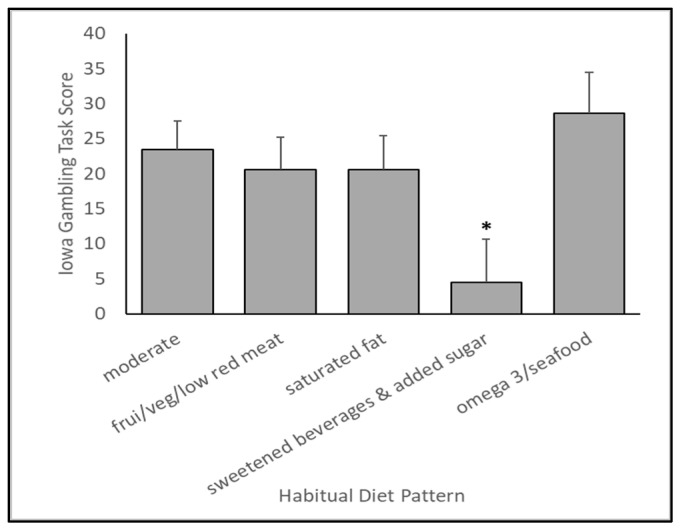
A diet pattern characterized by higher consumption of sweetened beverages and added sugar is associated with a lower Iowa Gambling Task performance, suggesting lower decision making in those habitually consuming liquid foods containing added sugar. * Sweetened beverages and added sugar diet pattern significantly differed from all other diet pattern groups (vs. moderate, *p* = 0.0030; vs. fruit/veg/low red meat, *p* = 0.0315; vs. saturated fat, *p* = 0.0221; vs. omega 3/seafood, *p* = 0.0010).

**Figure 3:**
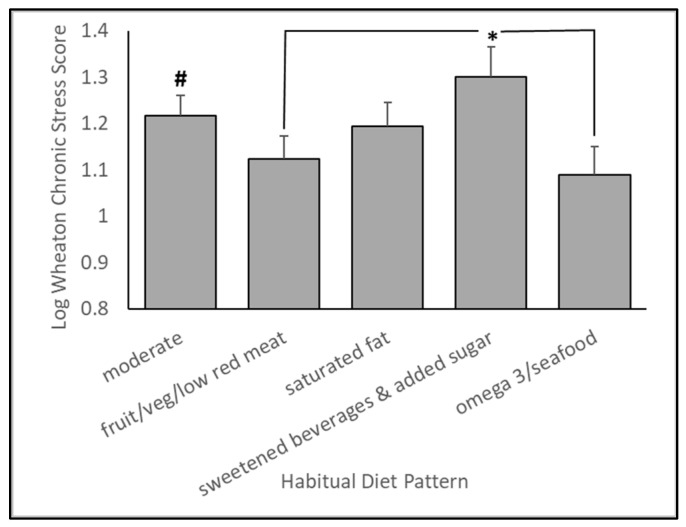
Diet patterns characterized by either higher consumption of omega 3 fatty acids or low in red meat and high in whole fruits and vegetables were associated with lower self-reported chronic stress exposure, suggesting lower chronic stress in those who typically consumed diets rich in omega 3 fatty acids and whole fruits and vegetables and low in red meat. * Diet pattern significantly differed from omega 3 (*p* = 0.0029) and fruit and fiber (*p* = 0.0048) patterns. **#** Diet pattern significantly differed from the omega 3 (*p* = 0.0178) and the fruit and fiber pattern (*p* = 0.0272).

As shown in Figure 1, five distinct diet patterns were identified by a cluster analysis. By visual inspection and through the use of a factor analysis, these patterns can be characterized as (1) generally moderate (solid black line, circles), (2) fruit and vegetables/low red meat (dotted black line, triangles), (3) saturated fat (solid gray line, circles), (4) sweetened beverages and added sugar (dashed black line, squares), and (5) omega 3 fatty acids and seafood (dashed gray line, squares). After statistically controlling for sex, age, BMI, education level, motor performance, and intelligence (WASI), diet pattern was significantly associated with Iowa Gambling Task performance (*p* = 0.0146), with an effect size (eta-square) of 0.0343. Likewise, diet pattern was also associated with self-reported chronic stress exposure (*p* = 0.0097), with an effect size (eta-square) of 0.0361. These results are shown in Figure 2 and Figure 3. These results suggest that, compared to all other identified diet patterns, habitual consumption of liquid foods containing added sugar is associated with poorer performance on the Iowa Gambling Task. Although our diet patterns were based on energy-adjusted intakes, we also tested whether total energy intake was associated with IGT performance. We did not find a statistically significant association (*p* = 0.7925) between total energy intake and performance in this affective decision-making task. Independently, diet patterns characterized by either a higher consumption of omega 3 fatty acids or low in red meat and high in whole fruits and vegetables were associated with lower self-reported chronic stress exposure, suggesting lower chronic stress in those who typically consumed diets rich in omega 3 fatty acids and whole fruits and vegetables and low in red meat.

To corroborate our findings based on the five distinct diet pattern clusters and to help quantifiably appreciate which food-related variables characterized the cluster patterns, we also used factor analysis of the same standardized dietary data. This statistical method reduces the large number of dietary variables into fewer dietary variables (factors) that can help with interpretation and visualization of the diet patterns (clusters). Using factor analysis, we found that a higher level of factor 5 (omega 3 and seafood) was associated (*p* = 0.0265) with lower levels of chronic stress exposure, which supports our findings using the cluster-derived diet patterns. Similarly, a higher level of factor 7 (sugary beverages/added sugar) was associated (*p* = 0.0015) with poorer performance in the Iowa Gambling Task. Additionally, we found that factor 13 (citrus/berries) was associated (*p* = 0.0222) with lower IGT performance, while a higher level of factor 10 (soy/isoflavones) was associated (*p* = 0.0226) with better performance in this executive function task. In Figure 1, the alternating shaded panels define the range of food variables on the *x*-axis that corresponded to each of the labeled factors. For example, factor 7 loaded most highly for sugary beverages; sweets; sugary beverages, including fruit juice; and added sugar. Within this group of food variables that represent factor 7, the cluster-derived sugar-sweetened beverage and added sugar diet pattern (black dotted line with solid squares) is highest in each of the sugary beverages, sweets, sugary beverages including fruit juice, and added sugar variables.

**Figure 4:**
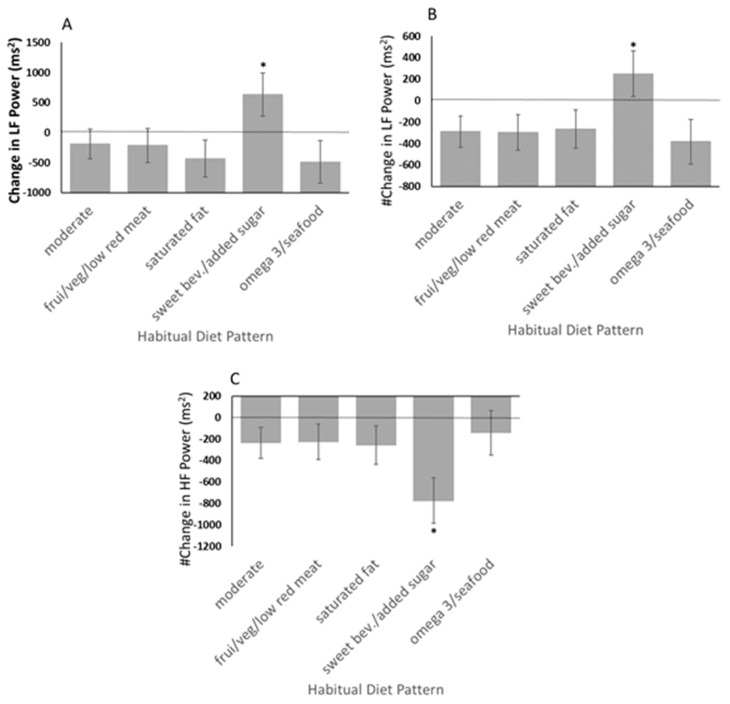
As a secondary objective, autonomic nervous system activity was examined as a possible mechanistic link between diet pattern and executive function. Heart rate variability was used to estimate ANS activity before and during the Iowa Gambling Task. (**A**) A diet pattern characterized by higher consumption of sweetened beverages and added sugar (sweet bev./added sugar) was associated with an increase in low frequency (LF) power during the Iowa Gambling Task relative to the pre-task level, suggesting that this diet pattern influenced a change in autonomic activity in response to performing the decision-making task. This same diet pattern was associated with an increase in LF power (**B**) and a decrease in high-frequency (HF) power (**C**) independent of any change in the total power as represented by the sum of LF and HF power. Therefore, this diet pattern may have reduced parasympathetic nervous system or vagal activity, as indicated by lower HF power, in response to performing this executive function task. * Sweetened beverages and added sugar diet pattern significantly differed from all other diet pattern groups (vs. moderate, *p* = 0.0030; vs. fruit/veg/low red meat, *p* = 0.0315; vs. saturated fat, *p* = 0.0221; vs. omega 3/seafood, *p* = 0.0010). # statistically normalized (adjusted) for change in the sum power of the HF and LF frequency bands. Dotted line represents no change.

Given the reported link between autonomic nervous system (ANS) activity and performance in the Iowa Gambling Task and other executive function tasks, we secondarily tested the effects of our cluster-identified diet patterns on ANS activity using heart rate variability (HRV) before and during task performance. As depicted in Figure 4, a diet pattern characterized by higher consumption of sweetened beverages and added sugar (A) was associated (*p* = 0.0249) with an increase in low frequency (LF) power HRV during the Iowa Gambling Task. The corresponding effect size (eta-square) was 0.0335. This same diet pattern was associated with an increase (*p* = 0.0374; eta-square = 0.0307) in LF power (B) and a decrease (*p* = 0.0374; eta-square = 0.0307) in high frequency (HF) power (C) independent of any change in the total power as represented by the sum of LF and HF HRV power. Furthermore, this diet pattern was associated (*p* = 0.0305) with a task-affiliated increase in the LF:HF ratio, which corresponded to an effect size (eta-square) of 0.0321.

## 4. Discussion

### 4.1. Influence of Diet Patterns on Iowa Gambling Task Performance

Our findings suggest that habitually consuming sugar-sweetened beverages along with a lower vegetable intake may negatively influence a group of cognitive functions related to decision making. We and others previously found that performance in the decision-making Iowa Gambling Task (IGT) was associated in some way with body weight regulation [4,5,6,7], and this may be related to diet [24]. While individual foods or nutrients can influence executive and other cognitive functions, our current results highlight a specific and whole diet pattern that may influence performance on this executive function task. Our findings also suggest that this influence of a habitual intake of sugar and a lower vegetable intake is independent of sex, age, education level, BMI, and sensorimotor ability. Elevated sugar consumption is generally thought to be detrimental to human health, and this may be particularly so when consumed in the liquid form [25]. Whether this extends to executive function in humans remains unknown, but there is some evidence to support this detrimental impact of sugar on cognitive function [26,27].

A study of adolescents did report an inverse association between sugar-sweetened beverage consumption and IGT performance in males, but not females [28]. However, that analysis targeted a priori specific food groups like sugar-sweetened beverages versus diet patterns. We show in adults that, compared to other diets also linked to poor health, such as those high in saturated fat, consuming a diet pattern high in liquid sugar may be particularly detrimental to affective decision making. Substance abuse was also shown to be associated with lower decision-making performance in the IGT [29]. Moreover, in an fMRI study, a habit of palatable food snacking that included highly sweetened food items was correlated with enhanced IGT-induced activity in the ventral striatum (reward) and lower IGT-induced activity in executive regions of the brain [30]. In that same study, vegetable consumption was associated with higher IGT-induced activity in pre-frontal regions that play a role in executive function. Thus, it is also possible that lower vegetable consumption and associated micronutrients in our cluster-identified sugar-sweetened beverage diet pattern (Figure 1, top panel—factor 2) partly explained the association of this diet pattern with lower IGT performance. As described by He et al. [30], neurological findings suggest a general personality-trait-related alteration in ventral striatal activity and reward sensitivity, which links to palatable food craving and overeating, as well as monetary reward. In an 8 week randomized control trial, habitual snacking on sweet and fatty foods was shown to be associated with decision-making brain circuitry and neurobehavioral alterations that were not explained by changes in body fat or other metabolic markers. In our study, which statistically controlled for BMI, we also found that total energy intake was not significantly associated with performance in the Iowa Gambling Task. Thus, energy- and body-weight-independent components of the total diet may play key roles in determining the status of brain circuitry related to higher cognitive functioning. Intervention studies using whole food diets are needed to directly test their ability to functionally affect these neurological pathways and corresponding affective decision making. Lastly, there are other possible mechanisms that could explain the negative association between lower IGT performance and the sugar-sweetened beverage and added sugar diet pattern. Inflammation, metabolic perturbations (e.g., insulin resistance), and/or shifts in neuronal plasticity in response to high sugar and poor-quality diets may affect higher order cognitive functioning, e.g., [27].

It is important to note that, while taken in isolation, factor 13 (citrus/berries/melon) was inversely associated with Iowa Gambling Task performance; the overall diet pattern (“fruit/veg/low red meat”), which was also highest in factor 13, was additionally highest in fiber, vegetables, and soy and lowest in red meat and saturated fat. This particular diet pattern, which tends toward vegetarian, was still significantly higher in Iowa Gambling Task performance than the pattern characterized by higher sweetened beverages, and no different from the other diet patterns. Thus, when taken as a whole pattern, multiple interacting dietary factors are key considerations when assessing the net effects of diet on cognitive function. Taken together, it seems that a pattern best characterized by higher sugar-sweetened beverage consumption is particularly influential on the gambling task performance since performance was lower compared to all other patterns (Figure 2). Since our findings are from a cross-sectional analysis, it is also possible that lower decision-making ability led to poorer dietary decisions and higher consumption of sugar-sweetened beverages. Future randomized control studies should be conducted to determine the decision-making effects of consuming whole food diets high in sugar-sweetened beverages.

### 4.2. Influence of Diet Patterns on Chronic Stress Exposure

In parallel, we also found an inverse association between chronic stress exposure and “fruit/veg/low red meat” and “omega 3/seafood” cluster-identified patterns (Figure 3). These findings indicate that chronic stress exposure and its perceived impact may be lower in people who regularly consume whole food diets rich in omega 3 fatty acids or high in fruits and vegetables. However, this seems to be especially true when compared to the sugar-sweetened beverage diet pattern, which was associated with a relatively higher reported chronic stress exposure. In support of our findings, we recently showed that a whole food diet intervention based on the Dietary Guidelines for Americans reduced perceived chronic stress in those participants who had increased vegetable consumption relative to their habitual diet before starting the intervention [3]. Other reports showed that fruit and vegetable consumption is associated with lower self-reported perceived stress [31,32,33,34,35]. Micronutrients in fruits and vegetables, such as the B vitamins and their metabolites, calcium, vitamin C, zinc, and magnesium, promote healthy regulation of brain pathways critical to maintaining a healthy mood and emotional state (see [3]). Although speculative, this may partly explain the observed association between the fruit and vegetable diet pattern and lower reported chronic stress.

Reports also show that consumption of foods higher in omega 3 fatty acids is associated with reduced chronic stress and physiological markers of stress under a variety of contexts [36,37,38,39]. Although stress has been linked to executive function degradation, including lower Iowa Gambling Task performance [40], our observations suggest that poor dietary habits affiliated with psychological stress (e.g., higher sugar consumption and lower fruit and vegetable consumption) may partly explain the known link between chronic stress and decision-making degradation. Additionally, given the apparent influence of diet pattern on chronic stress in this study, it is possible that chronic stress partly explained the observed association between diet pattern and Iowa Gambling Task performance. However, these were independent analyses and we cannot determine in this cross-sectional study diet-pattern-related mechanistic relationships between Iowa Gambling Task performance and chronic stress exposure. Our results do further support the importance of diet and stress interrelationships. More research is needed to test the mechanisms linking diet, stress, and executive function.

### 4.3. Influence of Diet Patterns on Autonomic Nervous System Activity

Autonomic nervous system regulation may be a key physiological effector of affective decision making, including decisions made during the Iowa Gambling Task [20,21,22,23]. We found that a diet pattern characterized by higher consumption of sweetened beverages and added sugar was associated with an increase in low-frequency (LF) power heart rate variability (HRV) during the Iowa Gambling Task, suggesting that this diet pattern may have altered autonomic activity in response to performing the decision-making task. This same diet pattern was associated with an increase in LF power and a decrease in high-frequency (HF) power independent of any change in the total HRV power as represented by the sum of LF and HF power. High-frequency HRV power is believed to represent activity in the parasympathetic nervous system [41] and supports a more reflective decision-making ability. While it remains to be entirely clear, LF power can result from both sympathetic (SNS) and parasympathetic (PNS) nervous system activity, and LF is thought to indicate baroreceptor reflex regulatory-related activity [41]. The ratio of LF to HF has been used to indicate the balance between the sympathetic and parasympathetic nervous system activity, with a higher ratio marking greater sympathetic tone [41]. However, the contributions of SNS and PNS to LF are difficult to discern and, thus, the interpretation of alterations in the LF:HF ratio remains controversial. Regardless, the observed associations between IGT-related changes in these HRV metrics and a diet pattern characterized by higher sugar-sweetened beverages and added sugar suggest a diet-related shift in autonomic response that is consistent with a reduced decision-making ability.

### 4.4. Overall Implications

This study provides further evidence for the potential detrimental relationship between decision making and a diet pattern predominantly characterized by high sugar-sweetened beverages and lower vegetable consumption. Our findings provide new information about diet patterns and affective decision making that might help to inform on key whole food diets that facilitate improved executive function independent of sex, age, BMI, and education level. As such, our results further support the potential importance of whole diet patterns on cognitive disease prevention.

### 4.5. Limitations

We acknowledge that there are limitations associated with this study. Dietary questionnaires based on participant recall are imperfect tools for measuring food intake and food intake habits. The cross-sectional nature of this data analysis is another potential limitation because we cannot assert causality or mechanisms of action. Whole food diet interventions are needed to better assess the impact and mechanisms of diet on executive functions affiliated with decision making. Furthermore, although we identified psychological and physiological variables that may in part link diet and decision making, prospective studies should be conducted to help explain mechanisms connecting certain diet patterns and decision making. We did not formally assess alertness, and although it is possible that differences in general alertness could have influenced associations between diet and cognitive performance, we did not visually observe any signs of a significant lack in alertness in the participants. It is possible that other factors, such as working memory, may influence executive function performance. However, this cognitive domain is an integral part of executive function and we did not include this measure in our statistical tests.

## Data Availability

Requests for data from the USDA ARS WHNRC individual Metabolism and Physiological Signatures and Phenotyping Studies used in this analysis should be made via an email to the senior WHNRC author on this publication. Requests will be reviewed by a committee consisting of the study investigators.
